# Effects of 650 nm laser acupuncture on cartilage, bone, and skeletal muscle in osteoarthritis

**DOI:** 10.1016/j.bonr.2025.101864

**Published:** 2025-07-31

**Authors:** Seung-Ho Seo, Sang-Mi Kang, Yang-Hee You, Chang-Su Na

**Affiliations:** a College of Korean Medicine, Dongshin University, 120-9 Dongsindae-gil, Naju, Republic of Korea

**Keywords:** Laser acupuncture, Osteoarthritis, Cartilage preservation, Bone morphometry, Muscle preservation

## Abstract

This study evaluated the therapeutic effects of 650 nm laser acupuncture at 10 mW and 20 mW in a monosodium iodoacetate (MIA) induced osteoarthritis (OA) rat model. OA was induced by intra-articular injection of MIA, and laser acupuncture was applied to GB34 and GB39 twice weekly for four weeks. Cartilage preservation was assessed by Safranin-O staining, pain by hind paw weight distribution, bone structure by micro-CT analysis of bone volume fraction, trabecular volume, and cortical thickness, and muscle condition by histology and wet weight of the gastrocnemius and quadriceps. Both laser treatments reduced cartilage degeneration and improved weight-bearing. The 10 mW group showed greater improvements than the 20 mW group, including higher proteoglycan content, better bone structural parameters, and greater muscle mass. These results indicate that 10 mW laser acupuncture is more effective than 20 mW in reducing joint damage and preserving musculoskeletal tissue. The findings support the use of low-power laser acupuncture as a non-invasive treatment for OA. The study also shows that higher laser power does not necessarily lead to better outcomes, highlighting the need for appropriate dose selection. Further studies are needed to assess long-term effects and investigate underlying mechanisms.

## 1 Introduction

Osteoarthritis (OA) is a chronic degenerative joint disease that significantly impacts global health, affecting over 500 million people worldwide (Kang et al., 2023; Lu et al., 2024). It primarily affects the knee, hip, and other weight-bearing joints, causing pain, stiffness, and reduced mobility (Fernández-Moreno et al., 2024). The management of OA involves a multifaceted approach, including non-pharmacological interventions such as exercise, weight management, and education (Mark et al., 2024). Pharmacological treatments focus on symptom management, with NSAIDs, acetaminophen, and intra-articular injections being common options (Fernández-Moreno et al., 2024; Mark et al., 2024). However, current treatments are often inadequate, highlighting a significant unmet need for effective therapies. The economic burden of OA is substantial, with high healthcare costs and productivity losses. The expected rise in OA prevalence due to an aging population and increasing obesity rates highlights the need for improved management strategies and potential disease-modifying treatments.

Laser acupuncture therapy (LAT) is an approach that combines traditional acupuncture principles with laser therapy. This technique uses low-intensity laser light to stimulate specific acupuncture points without the need for needle insertion, making it a non-invasive and painless alternative to conventional acupuncture (Chang et al., 2023; Di Francesco et al., 2024). LAT has gained attention in recent years as a safe and bloodless alternative to needle acupuncture, particularly for the treatment of various conditions, including arthritis (Trivedi et al., 2022; Di Francesco et al., 2024). The potential benefits of LAT in arthritis treatment are attributed to its anti-inflammatory, analgesic, circulatory, and regenerative effects (Zhang and Qu, 2023). However, it's important to note that research on LAT, particularly for arthritis treatment, is still in its early stages. While some studies have shown promising results, there is a need for more rigorous and comprehensive research to validate its efficacy fully.

This study aims to evaluate the therapeutic effects of 650 nm laser acupuncture therapy on key pathological features in a mono-iodoacetate (MIA) induced OA model, specifically focusing on its impact on cartilage degeneration, pain reduction, and the prevention of muscle atrophy. Through this investigation, we seek to clarify the potential of laser acupuncture as a non-invasive treatment approach for mitigating structural and symptomatic manifestations of OA.

## 2 Materials and methods

### 2.1 Experimental animals

Male Sprague-Dawley rats (9 weeks old, 270–300 g) were obtained from Samtaco Bio Korea. The animals were housed under controlled conditions (24 ± 2 °C, 12-h light/dark cycle) with ad libitum access to sterile drinking water and a standard pellet diet. They were acclimatized for one week prior to the experiment and monitored daily, with clinical records maintained throughout the study. All animal handling and experimental procedures were conducted in accordance with the ethical guidelines of the University of Dong-sin Animal Ethics Committee (Approval number: DSU2023-03-04).

### 2.2 Osteoarthritis induction and experimental grouping

A total of 24 rats were randomly assigned to four groups (*n* = 6 per group): normal (Normal), OA model (OA), OA treated with 10 mW laser acupuncture (OA-10 mW), and OA treated with 20 mW laser acupuncture (OA-20 mW). On day 0, OA was induced by a single intra-articular injection of 1.5 mg monosodium iodoacetate (MIA; Sigma-Aldrich, St. Louis, MO, USA) dissolved in 50 μL of sterile 0.9 % saline into the left knee joint. Rats in the OA, OA-10 mW, and OA-20 mW groups received the MIA injection, while those in the Normal group received an equal volume of saline (Wang et al., 2019).

### 2.3 Laser acupuncture treatment protocol

**Fig. 1:**
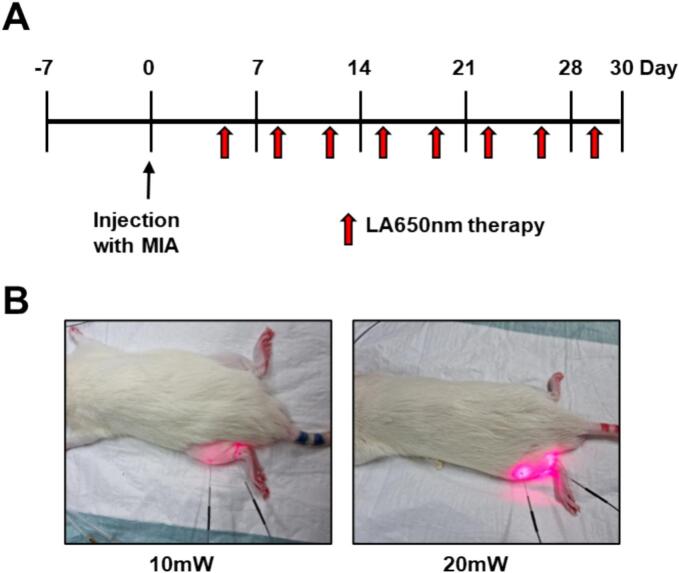
**Experimental design and laser acupuncture therapy setup.** (A) Timeline of mono-iodoacetate **(**MIA) injection and 650 nm laser therapy sessions. (B) Under anesthesia, a fiber laser needle was sequentially positioned at GB34 (Yangneungcheon) and GB39 (Hyeonjong) acupuncture points, with 10 mW/50 Hz and 20 mW/50 Hz laser settings applied for 3 min per site on the hind limbs of rats.

The experimental design is illustrated in Fig. 1. Specifically, laser acupuncture was administered twice per week for 4 weeks, beginning three days after MIA injection, resulting in a total of 8 treatment sessions. Laser acupuncture was performed using a 650 nm fiber-type diode laser system (Ellise; Wontech Co. Ltd., Daejeon, Korea) at 10 mW or 20 mW, 50 Hz, for 3 min (180 s) per acupoint. The 20 mW setting was selected based on our prior studies demonstrating its therapeutic efficacy in both clinical (chronic low back pain) and preclinical (musculoskeletal injury) models using the same device, while the 10 mW condition was included to explore the potential effectiveness of lower power settings and to investigate dose–response relationships in OA models (Kim et al., 2022). The laser beam was emitted from a fiber-integrated sterile acupuncture needle with a 0.2 mm diameter (spot size: 0.000314 cm^2^). Based on this, the power density was 31.8 W/cm^2^ (10 mW) and 63.7 W/cm^2^ (20 mW). The corresponding energy doses were 1.8 J and 3.6 J per point, with energy densities of 5732.5 J/cm^2^ and 11,465 J/cm^2^, respectively. These values align with manufacturer specifications (e.g., 38,216.56 J/cm^2^ at 20 mW for 10 min). Laser light was delivered via sterile, single-use acupuncture needles (outer diameter: 0.3 mm; length: 30 mm) integrated with optical fibers, enabling light emission from the needle tip inserted into the tissue. The insertion depth was approximately 3 mm, depending on the anatomical structure of the acupoint. During treatment, rats were anesthetized with 2 % isoflurane via nose cone to ensure immobilization and minimize discomfort. The fiber-optic laser needle was sequentially applied to acupuncture points GB34 (Yangneungcheon) (Zhang et al., 2021) and GB39 (Hyeonjong) (Lei et al., 2024). GB34 is located just below the head of the fibula on the lateral aspect of the hind limb, and GB39 is approximately 3 cm above the lateral malleolus, along the posterior border of the fibula.

Control animals (Normal and OA groups) were anesthetized and placed on the same apparatus for an equivalent duration without laser activation. All animals were sacrificed 30 days after MIA injection. Following sacrifice, left knee joints were carefully dissected, fixed in 4 % paraformaldehyde overnight, and stored at 4 °C until imaging. To minimize potential confounders, all procedures were conducted at the same time of day and under standardized conditions. The order of treatments and assessments was randomized, and cage positions were periodically rotated. Group assignment was determined using a random number table, and behavioral and histological evaluations were conducted by investigators blinded to group allocation.

### 2.4 Micro-CT

Micro-CT analysis was performed on the fixed, excised left knee joints using a high-resolution imaging system (Quantum GX2, PerkinElmer, Hopkinton, MA, USA). The hind limbs were fixed in 10 % formalin and positioned on the scanning platform. The X-ray source was set to a current of 200 μA and a voltage of 90 kVp, with a field of view (FOV) of 36 mm × 10 mm and a voxel size of 10 μm. Micro-CT imaging was visualized using the 3D viewer software within the Quantum GX system. Image processing involved semi-automatic segmentation using the Volume Edit tools in the Analyze software package (AnalyzeDirect, Overland Park, KS, USA). Images were then converted into 2D and 3D formats using RmCTGX image analysis software to visualize the cartilage area.

### 2.5 Safranin-O with fast green staining

Following the Micro-CT scan, the fixed joint tissue was sequentially decalcified and dehydrated, after which paraffin blocks were prepared. The paraffin-embedded tissue was sectioned into 8 μm-thick slices using a microtome (HM325, Thermo Scientific, Germany) and mounted on slides. Sections were deparaffinized, stained with Hematoxylin (Muto, Japan, 20032) for 10 min, and rinsed under running water for 10 min. They were then stained with 0.001 % Fast Green (Sigma, USA) for 5 min, treated with 1 % acetic acid for 10 s, and counterstained with 0.1 % Safranin O (Sigma, USA) for 5 min. Following thorough washing for 10 min, the sections underwent a dehydration process and were imaged using an Axioscan 7 slide scanner (ZEISS, Germany). Image analysis was conducted with ZEN 3.2 software (ZEISS, Germany).

### 2.6 Pain assessment (hind paw weight distribution)

To evaluate pain behavior in the rat knee joint, changes in hind paw weight distribution were used as an index of joint discomfort (Bove et al., 2003). Weight-bearing distribution between the left (osteoarthritic) and right (contralateral control) limbs was measured at 6, 9, 13, 16, 20, 23, 27, and, 30 days following MIA induced OA. Hind paw weight distribution was recorded using a Paw Pressure Analgesia Meter 600MR (IITC Life Science, California, USA). Each rat was placed in a chamber positioned at a 60-degree angle so that each hind paw rested on a separate force plate. The force exerted by each hind limb was averaged over a 5-s period, with each data point representing the mean of three 5-s readings. Results are presented as the percentage difference in weight-bearing between the right (control) and left (osteoarthritic) limbs, calculated as: (weight of left / weight of right) × 100.

### 2.7 Bone histomorphometry analysis

Subchondral trabecular bone was analyzed from the reconstructed micro-CT cross-sectional images. Regions of interest (ROIs) with irregular anatomical contours were drawn for the medial and lateral tibial plateaus using CT Analyzer software (V 1.8.05, Skyscan, Kontich, Belgium). The volume of interest (VOI) included the subchondral trabecular bone starting below the subchondral plate and extending toward the growth plate, excluding cortical bone and the growth plate interface. Morphometric parameters were calculated by segmenting the images using a uniform threshold as described previously (Perilli et al., 2010). The following three-dimensional (3D) parameters were calculated for the medial VOI, lateral VOI, and total VOI (medial + lateral): bone volume (BV, mm^3^), bone volume fraction (BV/TV, %), trabecular volume (Tb.V, mm^3^), cortical bone area (Ct.Ar, mm^2^), and average cortical thickness (Ct.Th, mm). Bone morphometry was limited to the tibia, as the tibial plateau exhibits consistent degenerative changes in the MIA induced OA model. Measurements from the medial and lateral plateaus were averaged for group comparisons, as side-to-side differences were not the focus of this study.

### 2.8 Muscle cross-sectional area (CSA)

The gastrocnemius (GA) and quadriceps femoris (QF) muscles from the left hind limbs were excised and weighed (Cunha et al., 2019). The proximal fragments were fixed in 10 % formalin for 3 days, then embedded in paraffin. Muscle sections were cut into 6 μm slices and stained with hematoxylin (Muto, Japan, 20032) and eosin (Muto, Japan, 32002). The stained slices were imaged using a 3D HISTECH slide scanner (Pannoramic SCAN II, England). Myofiber cross-sectional area (CSA) was determined by measuring the muscle fiber diameter. Ten images were taken per muscle per rat using Slideviewer, with 20 fibers measured per image, totaling 200 myofibers measured per muscle.

### 2.9 Statistical analysis

All statistical analyses were performed using one-way ANOVA followed by Tukey's post hoc test to determine significant differences among the four experimental groups (Normal, OA, OA-10 mW, and OA-20 mW). Data were tested for normality using the Shapiro-Wilk test prior to ANOVA. The *p*-values presented in the figures reflect results from post hoc pairwise comparisons. A p-value less than 0.05 was considered statistically significant. All analyses were conducted using GraphPad Prism 9 (GraphPad Software, San Diego, CA, USA).

## 3 Results

### 3.1 Effects of laser acupuncture on cartilage integrity and pain reduction in an osteoarthritis model

**Fig. 2:**
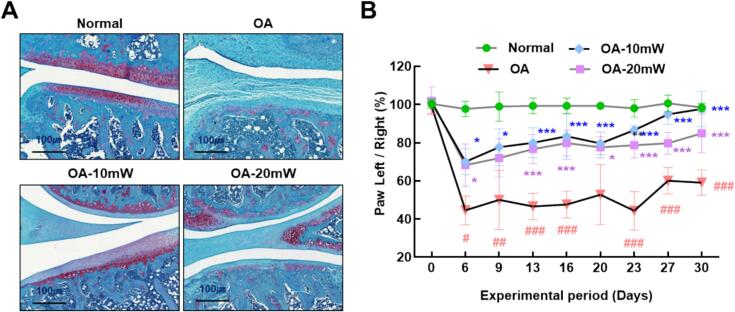
**Effects of 650 nm laser acupuncture on cartilage preservation and pain reduction in an mono-iodoacetate (MIA) induced osteoarthritis (OA) rat model.** (A) Representative histological images of knee joint cartilage stained with Safranin-O and Fast Green. The OA group shows marked cartilage degradation compared to the Normal group, while laser-treated groups (OA-10 mW and OA-20 mW) exhibit varying degrees of cartilage preservation. (B) Changes in hind paw weight distribution over 30 days, used as an indicator of joint pain. The OA group exhibited significantly impaired weight-bearing compared to the Normal group, while both laser-treated groups showed improvements, particularly the OA-10 mW group. Data are presented as mean ± SD (*n* = 6 per group). Statistical analysis was performed using one-way ANOVA followed by Tukey's post hoc test. #*p* < 0.05, ##*p* < 0.01, ###*p* < 0.001 vs. Normal group; *p < 0.05, **p < 0.01, *p < 0.001 vs. OA group.

Fig. 2A shows the effects of 10 mW and 20 mW laser acupuncture on cartilage preservation, as observed with Safranin-O and Fast Green staining. Both laser-treated groups (OA-10 mW, OA-20 mW) exhibited reduced cartilage degeneration compared to the untreated OA group. The laser treatments preserved proteoglycan content, indicated by stronger Safranin-O staining, with the cartilage structure resembling that of the normal control group. Fig. 2B illustrates changes in hind paw weight distribution over 30 days, demonstrating significant improvement in weight-bearing in the laser-treated groups. Both 10 mW and 20 mW laser treatments led to better weight distribution, reflecting pain reduction, with 10 mW treatment showing a more pronounced effect.

### 3.2 Effects of laser acupuncture on joint structure and bone morphometry in an osteoarthritis model

Our study aimed to evaluate the therapeutic effects of 650 nm laser acupuncture at different power levels (10 mW and 20 mW) on joint structure, meniscal ossification, and subchondral bone morphology in a MIA induced OA rat model. Specifically, we sought to assess whether laser acupuncture could mitigate joint degeneration, reduce meniscal ossification, and improve subchondral bone parameters.

**Fig. 3:**
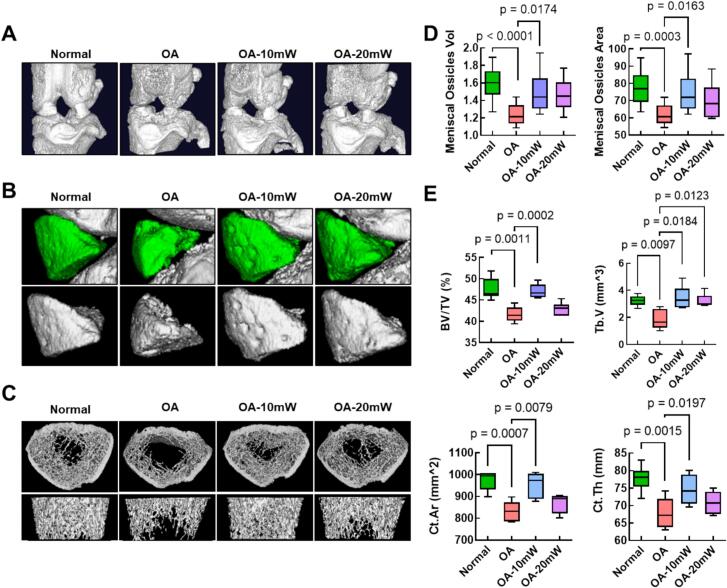
**Effects of 650 nm laser acupuncture on joint structure, meniscal ossification, and subchondral bone morphology in a mono-iodoacetate (MIA) induced osteoarthritis (OA) rat model.** (A) Three-dimensional micro-CT images of knee joints across the normal, untreated OA, 10 mW laser-treated OA, and 20 mW laser-treated OA groups, showing structural differences in joint integrity. (B) Three-dimensional images of meniscal ossicles in the knee joint, illustrating differences in ossicle size among the groups. (C) Cross-sectional micro-CT images of subchondral bone, highlighting differences in trabecular and cortical structures between the groups. (D) Quantitative analysis of meniscal ossicle volume and area shown in (B), with 10 mW laser-treated group demonstrating a statistically significant reduction in ossicle size compared to the untreated OA group (*p* = 0.0002 for volume and *p* = 0.0011 for area). (E) Quantitative analysis of subchondral bone parameters shown in (C), including bone volume fraction, trabecular volume, cortical bone area, and cortical bone thickness. The 10 mW laser-treated group shows significant improvements in bone volume fraction (*p* = 0.0079), trabecular volume (*p* = 0.0007), cortical bone area (*p* = 0.0197), and cortical bone thickness (*p* = 0.0015) compared to the untreated OA group, while 20 mW group shows no statistically significant changes in these parameters. Box-and-whisker plots were used to illustrate the distribution and variability of the data. In these plots, the center line indicates the median, the box edges represent the 25th and 75th percentiles, and the whiskers correspond to the minimum and maximum values.

Fig. 3A presents three-dimensional micro-CT images of the knee joints for the different groups: normal, untreated OA, 10 mW laser-treated OA, and 20 mW laser-treated OA. The untreated OA group shows notable joint structure deterioration compared to the normal group. In contrast, the laser-treated groups, especially 10 mW group, exhibit less joint degradation, suggesting a protective effect of laser acupuncture on joint integrity. Fig. 3B provides images of meniscal ossicles in the knee joint, focusing on ossicle structures within each group. The ossicles in the untreated OA group appear larger and more pronounced compared to those in the normal and laser-treated groups. 10 mW laser-treated group shows a visible reduction in meniscal ossicle size compared to the untreated OA group, while 20 mW group exhibits a lesser degree of reduction. Fig. 3C displays cross-sectional micro-CT images of the subchondral bone in each group, highlighting differences in trabecular and cortical structure. The untreated OA group shows considerable deterioration in trabecular bone structure compared to the normal group, with sparse and degraded trabeculae. In contrast, 10 mW laser-treated group shows a denser trabecular structure, indicating a preservation effect on bone integrity. 20 mW laser-treated group, however, shows less improvement compared to 10 mW group. Fig. 3D provides quantitative data on meniscal ossicle volume and area, corresponding to the images in Fig. 3B. 10 mW laser-treated group demonstrates a statistically significant reduction in meniscal ossicle volume (*p* = 0.0002) and area (*p* = 0.0011) compared to the untreated OA group. In 20 mW group, no significant reduction is observed, as indicated by the lack of *p*-values. Fig. 3E and Fig. 3F provide quantitative analyses of subchondral bone parameters shown in Fig. 3C. Fig. 3E shows improvements in bone volume fraction (*p* = 0.0079) and trabecular volume (*p* = 0.0007) in 10 mW laser-treated group compared to the untreated OA group, while 20 mW group does not show significant improvement in these parameters. Fig. 3F displays cortical bone parameters, including cortical bone area (*p* = 0.0197) and cortical thickness (*p* = 0.0015), again showing significant improvement in 10 mW laser-treated group, with no statistically significant changes observed in 20 mW group.

### 3.3 Effects of laser acupuncture on muscle mass and cross-sectional area in an osteoarthritis model

**Fig. 4:**
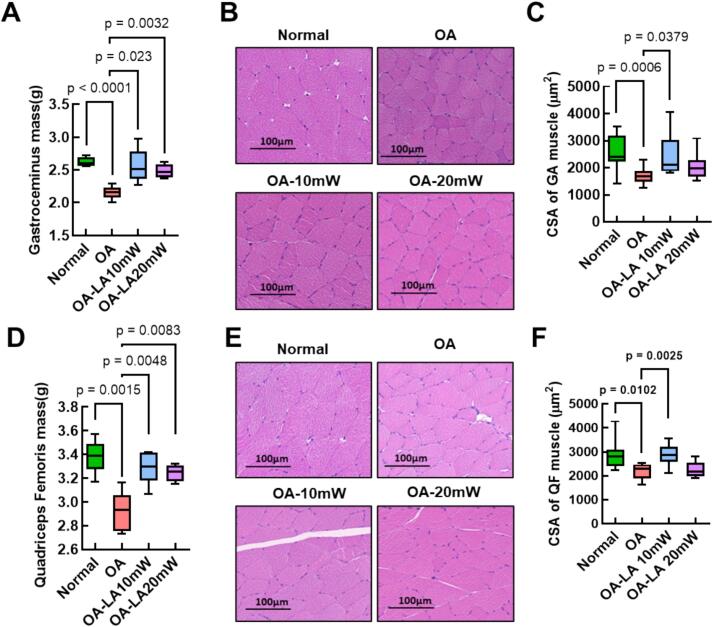
**Effects of 650 nm laser acupuncture on muscle mass and muscle fiber cross-sectional area in an mono-iodoacetate (MIA) induced osteoarthritis (OA) rat model.** (A, D) Wet weight measurements of the gastrocnemius (GA) and quadriceps femoris (QF) muscles across the normal, OA, 10 mW laser-treated OA, and 20 mW laser-treated OA groups. Muscle mass was significantly reduced in the OA group compared to the normal group, while both 10 mW and 20 mW laser-treated groups showed increased muscle weight, particularly in 10 mW group. (B, E) Representative Hematoxylin and Eosin (H&E) stained images of GA and QF muscle sections from each group, illustrating structural differences in muscle fiber integrity. The OA group exhibits muscle atrophy and reduced fiber size compared to the normal group, with laser-treated groups showing improved muscle structure, especially at 10 mW. (C, F) Quantitative analysis of cross-sectional area (CSA) of muscle fibers in GA and QF muscles. The CSA is significantly decreased in the OA group, whereas both laser-treated groups demonstrate preservation of muscle fiber area, with 10 mW group showing the most pronounced effect. Box-and-whisker plots were used to illustrate the distribution and variability of the data. In these plots, the center line indicates the median, the box edges represent the 25th and 75th percentiles, and the whiskers correspond to the minimum and maximum values.

Laser acupuncture at 10 mW and 20 mW effectively attenuated muscle atrophy in both the gastrocnemius and quadriceps femoris muscles, as indicated by increased muscle mass and preserved cross-sectional area. Fig. 4A and Fig. 4D present the wet weight measurements of the gastrocnemius and quadriceps femoris muscles, respectively, across all groups. Both muscles showed a significant reduction in mass in the OA group compared to the normal group, indicating muscle atrophy associated with OA. Laser acupuncture at 10 mW significantly improved both gastrocnemius and quadriceps muscle mass and fiber area, showing more consistent effects than the 20 mW treatment, which exhibited variable outcomes. Fig. 4B and Fig. 4E display representative Hematoxylin and Eosin-stained images of gastrocnemius and quadriceps femoris muscle sections, respectively. In contrast, both laser-treated groups display improved muscle fiber integrity, with fibers appearing more similar to those in the normal group, particularly in 10 mW group. Fig. 4C and Fig. 4F provide quantitative analyses of the cross-sectional area of muscle fibers in the gastrocnemius and quadriceps femoris muscles, respectively. The cross-sectional area is significantly reduced in the OA group, reflecting muscle atrophy. Both laser-treated groups, especially 10 mW group, show a preservation of muscle fiber area, indicating a protective effect of laser acupuncture against muscle degradation.

## 4 Discussion

The aim of this study was to evaluate the therapeutic effects of 650 nm laser acupuncture at different power levels (10 mW and 20 mW) in mitigating the structural and symptomatic impairments associated with OA in an MIA induced rat model. Specifically, we focused on assessing the impact of laser acupuncture on cartilage preservation, pain reduction, and bone morphology, including subchondral bone structure and meniscal ossification. To address these objectives, we employed a combination of histological analyses, pain behavior tests, and advanced imaging techniques, including 3D micro-CT. Laser acupuncture reduced cartilage degeneration, as shown by enhanced proteoglycan retention in Safranin-O staining. Additionally, laser treatments led to improved weight-bearing ability, suggesting a reduction in pain. Importantly, laser acupuncture also mitigated subchondral bone degeneration, as indicated by improved bone morphometric parameters, including bone volume fraction, trabecular volume, and cortical thickness. Overall, these findings indicate that 650 nm laser acupuncture at 10 mW may offer a promising non-invasive approach to treating OA-related structural damage and pain.

Our findings align with existing evidence that acupuncture-based therapies confer both symptomatic relief and structural protection in OA. Prior studies have shown that both laser acupuncture (LA) and electroacupuncture (EA) can alleviate joint pain and improve functional outcomes in knee OA patients (Trivedi et al., 2022). In particular, the observed preservation of proteoglycan content aligns with existing evidence that acupuncture treatments can minimize extracellular matrix degradation and protect cartilage structure (Ye et al., 2023). Notably, our results also indicate that the 10 mW treatment group showed more consistent improvements in cartilage integrity, muscle mass, and subchondral bone preservation than the 20 mW group. This observation suggests that the therapeutic effects of laser acupuncture may not follow a strictly linear dose-response relationship, warranting further investigation into optimal power settings for OA management.

The therapeutic differences observed between the 10 mW and 20 mW groups in this study may be related to the dose–response characteristics of photobiomodulation (PBM). Although the precise mechanisms remain to be fully elucidated, previous studies suggest that PBM may exhibit a biphasic dose–response pattern, where an optimal therapeutic window exists between insufficient and excessive irradiation levels (Huang et al., 2009; Hamblin, 2017). In this context, the relatively greater effectiveness of 10 mW compared to 20 mW across parameters such as cartilage preservation, bone morphometry, and muscle maintenance suggests that the lower power may have fallen closer to the optimal therapeutic window in our model.

Furthermore, the use of an invasive laser acupuncture system likely contributed to this outcome by enabling more efficient light delivery directly to the target tissues. Unlike transcutaneous approaches, the invasive method reduces energy loss due to reflection or scattering at the skin surface, thereby increasing the effective dose at the cellular level even when using low-output power settings. These findings provide a rationale for further exploration of low-dose, tissue-targeted PBM strategies in joint disorders, and suggest that optimizing delivery methods may be as important as adjusting output power in achieving therapeutic efficacy.

Additionally, the observed cartilage preservation and pain reduction may involve multiple mechanisms, including the modulation of inflammatory pathways. Acupuncture is known to inhibit cytokine production via signaling pathways such as p38 MAPK and to regulate mitochondrial responses that limit chondrocyte apoptosis (Jang et al., 2024; Spittler et al., 2025). The choice of GB34 and GB39 as stimulation sites may have further contributed to these effects, given their established roles in musculoskeletal and joint disorders. GB34, categorized as a tendon influential point, has been shown to reduce inflammatory cytokines (e.g., IL-1β, TNF-α) and protect against chondrocyte apoptosis (Yan et al., n.d.). GB39, identified as a bone influential point, modulates synovial angiogenesis and joint inflammation through the HIF-1α/VEGF pathway (Zhu et al., 2019). Thus, their combined stimulation may provide synergistic effects in suppressing inflammation, preserving cartilage integrity, and alleviating OA-related symptoms (Chen et al., 2014; Wei et al., 2024).

Based on the experimental results, laser acupuncture demonstrates significant positive effects on joint structure and bone morphometry in OA. The improvements in bone volume fraction, trabecular volume, cortical bone area, and cortical thickness suggest that laser acupuncture may help mitigate bone degradation associated with OA (Huang et al., 2024). This aligns with previous research indicating that acupuncture can affect structural aspects of OA, potentially by modulating inflammatory factors and regulating cytokines to limit chondrocyte death (Ruan et al., 2021; Huang et al., 2024). The reduction in meniscal ossification observed in the laser-treated groups is particularly noteworthy, as it suggests that laser acupuncture may help preserve joint integrity. These findings contribute to the growing body of evidence supporting acupuncture as a non-pharmacological treatment for OA, addressing not just symptoms but also underlying structural changes (Jankaew et al., 2023; Huang et al., 2024). The results suggest that laser acupuncture may partially attenuate muscle atrophy in the gastrocnemius and quadriceps femoris muscles, which could contribute to musculoskeletal support in the context of OA. This is particularly important as muscle atrophy is a common complication in OA that can exacerbate joint instability and pain (Jankaew et al., 2023).

At the cellular level, laser therapy has been shown to facilitate the proliferation of myosatellite cells and enhance angiogenesis and formation of myotubes. This leads to increased regeneration of muscle fibers and higher density of mitochondria, which may explain its effects on muscle preservation (Jankaew et al., 2023). These findings support the potential of laser acupuncture as a comprehensive treatment approach for OA, addressing not only joint and bone health but also surrounding muscle tissue and underlying inflammatory processes.This multifaceted effect could be particularly beneficial in maintaining overall joint function and mobility in OA patients.

This study offers several advantages over previous research, including a comprehensive evaluation of muscle mass and cross-sectional area across multiple muscle groups affected by OA. The use of histological imaging provided valuable insights into muscle fiber integrity, enhancing our understanding of how laser acupuncture may contribute to muscle preservation. However, certain limitations should be noted. The sample size, while sufficient for this study, may be smaller than optimal for detecting broader therapeutic effects, potentially limiting the generalizability of the findings. Additionally, this study focused on the immediate effects of laser acupuncture, with no long-term follow-up, preventing conclusions regarding the duration of the observed benefits. Moreover, although cartilage was qualitatively assessed using Safranin-O and Fast Green staining, standardized scoring systems such as the OARSI score were not applied. Future studies may benefit from incorporating such scoring methods to enhance histological evaluation. Furthermore, although muscle atrophy and pain-related behaviors were both evaluated, this study did not include a correlation analysis between these parameters. Future studies incorporating such analysis may help clarify the mechanistic relationship between muscle degradation and functional outcomes in OA. Future research should aim to increase sample size, incorporate long-term follow-up, explore combination therapies, and further investigate the optimal dosing parameters for laser acupuncture. Despite these constraints, this study contributes valuable insights into the potential of laser acupuncture for muscle preservation in OA, providing a solid foundation for further exploration in this promising area.

## 5 Conclusion

This study evaluates the therapeutic effects of 650 nm laser acupuncture at 10 mW and 20 mW on cartilage preservation, pain reduction, bone structure, and muscle mass in an MIA induced osteoarthritis rat model. The results show that laser acupuncture significantly reduces cartilage degeneration, preserves proteoglycan content, and improves weight-bearing ability, suggesting pain relief. Furthermore, it mitigates subchondral bone degeneration, as evidenced by improved bone morphometric parameters, and helps preserve muscle mass, particularly in the gastrocnemius and quadriceps femoris muscles. Among the two power settings, the 10 mW treatment consistently showed superior outcomes across multiple parameters compared to the 20 mW treatment. These findings suggest that low-level laser acupuncture, particularly at 10 mW, could be a promising non-invasive treatment for osteoarthritis.

## CRediT authorship contribution statement

**Seung-Ho Seo:** Writing – original draft, Software, Methodology, Data curation. **Sang-Mi Kang:** Data curation. **Yang-Hee You:** Investigation. **Chang-Su Na:** Writing – review & editing, Project administration, Methodology.

## Declaration of competing interest

The authors declare that they have no conflicts of interest.

## Data Availability

Data will be made available on request.
